# Segregation and Integration of Auditory Streams when Listening to Multi-Part Music

**DOI:** 10.1371/journal.pone.0084085

**Published:** 2014-01-24

**Authors:** Marie Ragert, Merle T. Fairhurst, Peter E. Keller

**Affiliations:** 1 Max Planck Institute for Human Cognitive and Brain Sciences, Research Group: Music Cognition and Action, Leipzig, Germany; 2 Max Planck Institute for Human Cognitive and Brain Sciences, Research Group: Early Social Development, Leipzig, Germany; 3 The MARCS Institute, Music Cognition and Action Group, University of Western Sydney, Sydney, Australia; Baycrest Hospital, Canada

## Abstract

In our daily lives, auditory stream segregation allows us to differentiate concurrent sound sources and to make sense of the scene we are experiencing. However, a combination of segregation and the concurrent integration of auditory streams is necessary in order to analyze the relationship between streams and thus perceive a coherent auditory scene. The present functional magnetic resonance imaging study investigates the relative role and neural underpinnings of these listening strategies in multi-part musical stimuli. We compare a real human performance of a piano duet and a synthetic stimulus of the same duet in a prioritized integrative attention paradigm that required the simultaneous segregation and integration of auditory streams. In so doing, we manipulate the degree to which the attended part of the duet led either structurally (attend melody vs. attend accompaniment) or temporally (asynchronies vs. no asynchronies between parts), and thus the relative contributions of integration and segregation used to make an assessment of the leader-follower relationship. We show that perceptually the relationship between parts is biased towards the conventional structural hierarchy in western music in which the melody generally dominates (leads) the accompaniment. Moreover, the assessment varies as a function of both cognitive load, as shown through difficulty ratings and the interaction of the temporal and the structural relationship factors. Neurally, we see that the temporal relationship between parts, as one important cue for stream segregation, revealed distinct neural activity in the planum temporale. By contrast, integration used when listening to both the temporally separated performance stimulus and the temporally fused synthetic stimulus resulted in activation of the intraparietal sulcus. These results support the hypothesis that the planum temporale and IPS are key structures underlying the mechanisms of segregation and integration of auditory streams, respectively.

## Introduction

Multi-part music is an example of a complex auditory scene. Bregman [1] has proposed that stream segregation and, through it, auditory scene analysis is based on general gestalt principles such as temporal proximity or closeness in pitch. Through these principles, stream segregation for multi-part music is based for example, on distances in pitch space, with small distances belonging to the same musical part and large distances between pitches allowing for differentiation of parts (for more details on segregation cues in music see [2], [3]). Another grouping cue that has been proposed is a hierarchical structural relationship of melody and accompaniment, with the melody dominating perceptually over the harmonizing accompaniment [1], [4], [5]. However, segregating music into its component streams is often made more challenging by different parts having the same or similar timbre (e.g. string quartet or piano duets) and harmony between the parts as horizontal (i.e. over time) and vertical (i.e. fusion of tones within chords) grouping may compete for perception [1], [6], [7]. Temporal components such as differences in note onsets or asynchronies between parts might represent more reliable cues in such situations [1], [6], [8].

The perceptual analysis of complex auditory scenes relies upon two specific mechanisms, stream segregation and stream integration. While stream segregation is necessary to group sequential auditory information coming from different sources, integration, as a higher order process, then places streams into the same representational space to allow for an assessment of the relationship between them (i.e. distance, space, structural importance) [9]–[11]. Two neuroanatomical structures have been implicated in these mechanisms.

It has been proposed that the planum temporale (PT) is involved in segregating incoming auditory streams [12], [13]. More specifically, different relevant information about stimulus attributes such as spatial position, movement [13], temporal cues [14], [15] or general spectro-temporal patterns are used to segregate streams and are then used to forward stimulus information to the parietal lobe for further processing [12], [13].

The integration of information from different sources, on the other hand, is achieved through the involvement of the inferior parietal cortex (IPC). Across sensory modalities, the IPC has been implicated in the processing of the relationship [10] or magnitude [9], [16] of and between objects. Relevant to the auditory domain, this brain area has been shown to be activated during the assessment of pitch relations such as comparing a melody to a reversed melody [10], [17], [18] or the assessment of temporal relations (i.e. comparing time intervals, [19]) [19]–[22].

It has been hypothesized that a form of divided attention, termed “prioritized integrative attention” is employed when listening to or producing multi-part music [11], [23]–[25]. This kind of attention allows the listener to prioritize one of the streams while still integrating the rest so as to capture a holistic sound scape and to assess the relationships between the parts. Prioritized integrative attention may thus be uniquely suited to the investigation of auditory scene analysis, where both segregation and integration of streams is required.

Relationships between streams can be determined based on different attributes of the streams (i.e. louder than, higher in pitch than, faster than, etc.) and are especially important in music [26] as they contribute to the perception of a “conversation like” relationship between voices of instruments (cf. [27]). This relationship may be more abstract, encompassing, for example, leader and follower roles between the different instrument parts [28]. Leading and following in music can be described on a temporal basis: one player intentionally or unintentionally produces sounds slightly temporally ahead and, as such, is temporally leading [28]–[32]. Alternatively, leading and following can also be defined structurally with the melody leading and the accompaniment following, as is conventionally the case for many western styles of music [1], [4], [5], [11], [27], [33]. A hierarchy in which the melody leads or even dominates the accompaniment perceptually is sometimes considered to be analogous to visual figure-ground perception, with the melody defining the figure and the accompaniment the background [4], [5]. In everyday life, music listeners are in general more familiar with this kind of structural relationship (melody lead) than the reverse (accompaniment lead), which can influence their perception via top-down mechanisms [11]. Leader and follower roles can thus be defined either through a temporal manipulation, which relates to asynchronies between voices, or through the structural relationship of a musical piece, which relates to a hierarchical structure where the melody leads in western music.

In a recent paper [11] we were able to show that both kinds of relationship (structural and temporal) interact on a behavioral as well as on a neural level, highlighting the value of prioritized integrative attention tasks for ongoing research in music perception. The previous study explored the interaction of the leader-follower relationship factors by manipulating the temporal relationship and contrasting a natural performance stimulus without a global leader with an exaggerated global temporal leader. In this case the exaggeration, although synthetically created, was still within the range of natural performance asynchronies. The effect of the temporal relationship on behavioral as well as neural responses could however not be interpreted strongly in favor of the segregation mechanism, as both kinds of stimuli could be segregated on the basis of temporal cues. In the present study, the same task was used in order to explore in greater detail the neural underpinnings of segregation and integration as mechanisms involved in listening to multi-part music (piano duets). The leader-follower relationship was manipulated by using a recording of a real performance of the duet, which included natural local temporal variations between parts (asynchronies) and was contrasted with a synthetically computer-generated version of the duet in which there were no temporal variations within or between parts. The use of a synthetic control stimulus is consistent with common practice in imaging studies exploring the neural underpinnings of music listening, which employ synthetic stimuli instead of natural performances e.g. [34]. Participants were cued to follow (prioritize) one of two duet streams and therefore to segregate the streams present in a piano duet stimulus. A question about the leader-follower relationship between parts of the duet presented after the listening task, however, also necessitated participants to concurrently integrate the second stream into a common representational space with the first stream. Participants were required to judge whether the attended part was leading or following compared to the second duet part. Only by integrating the two streams could a picture of a leader-follower relationship between melody and accompaniment of the duet emerge.

In the performance stimulus, depending on the direction of the asynchrony, either the melody or the accompaniment part was temporally leading or following locally, but not globally across the entire recording (i.e., the median asynchrony between parts was close to zero). As such, there was no temporal relationship cue available for segregating the two piano duet streams. Both parts of the piano duet had the same instrumental timbre, therefore segregation of streams for both kinds of stimuli differed based on the temporal relationship between parts [1], [8]. The temporal relationship between parts, being one possible factor defining leader-follower roles in music, was expected to be a factor driving the perception of the leader-follower relationship between parts. Nevertheless, it was unclear whether the temporally separated performance stimulus or the - due to the lack of a changing temporal relationship between parts – much simpler temporally fused synthetic stimulus would be more difficult to judge.

For the subjective assessment of the leader-follower relationship, we thus posited that the performance stimulus could be rated based on its temporal relationship, its structural relationship or, as participants were not directly aware of these two components, a combination of both relationship factors. By contrast, the leader-follower relationship between parts of the synthetic stimulus could only be based on the structural, hierarchical relationship. A comparison of the two different stimulus types would thus shed light on the integration of the structural and temporal relationship factors between parts as well as on segregation processes based on the difference in the cues of the temporal relationship, which we hypothesized to involve the PT. The assessment of the relationship and thus integration of parts, however, was expected to be represented by common activations for both stimulus types – the temporally separable performance and temporally inseparable synthetic stimulus - within the IPC.

## Methods

### 2.1 Participants

Seventeen (8 female) right-handed healthy musicians with a mean age of 26.12 years (SD±4.2) volunteered to participate in this study. As described in our previous article [9], participants were experienced pianists with an average of 16.44 (SD±5.92) years of playing experience (except for one who was a musician with 10 years of clarinet and guitar experience) and had had no prior neurological or psychiatric disorders. All participants met the inclusion criteria for magnetic resonance (MR) experimentation and signed a written informed consent form to participate in the experiment according to the declaration of Helsinki as part of Max Planck Institute protocol. The experiment was approved by the ethics committee of the University of Leipzig. Participants were recruited from the Max Planck Institute for Human Cognitive and Brain Sciences' data base and were paid for their participation.

### 2.2 Design & Stimuli

**Figure 1:**
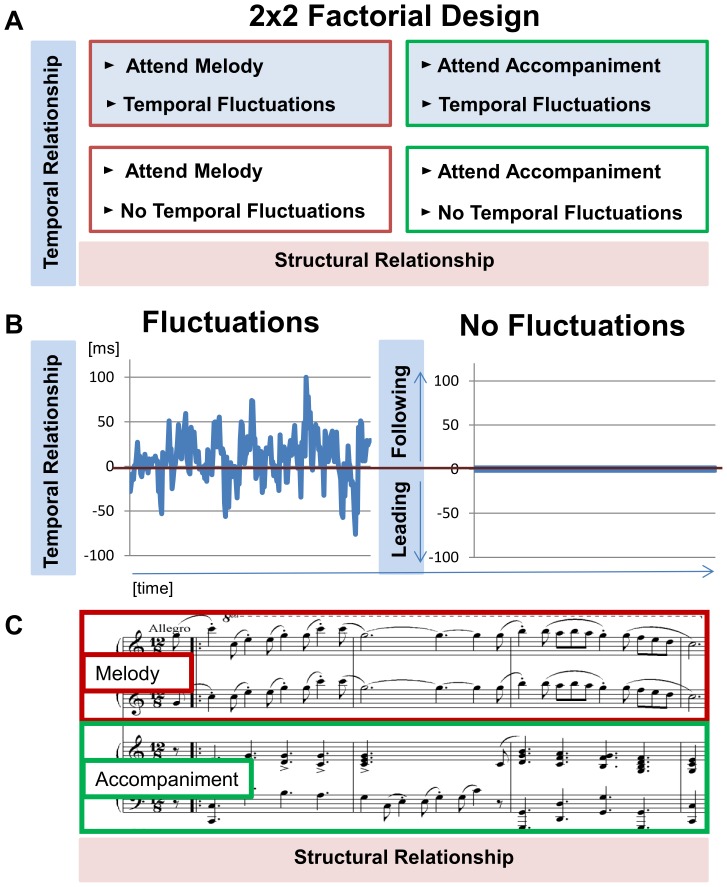
Factorial design. Factorial design with (A) the factors *temporal relationship* (temporal fluctuations vs. no temporal fluctuations) and *structural relationship* (red: attend melody vs. green: attend accompaniment). (B) Temporal relationship. On the left are depicted temporal fluctuations (asynchronies, in ms) of the performance stimulus over time, showing how the melody alternates between leading and following. On the right, there are no temporal fluctuations in the synthetic stimulus, perfect synchrony; no alternating roles of leading and following, as indicated by the straight line. (C) Structural relationship. Musical notation shows the beginning of the musical piece used for the stimuli. Both parts describe a clear hierarchical structure of a melody and an accompaniment consisting of harmonic chord progressions. The melody remains within a higher pitch range than the accompaniment, and in this sense its own auditory stream, throughout the stimulus. The red rectangle represents the task of prioritizing the melody; the green rectangle represents the task of attending to the accompaniment.

A 2×2 factorial design (Fig. 1A) was used to manipulate attention to the “structural relationship” and the “temporal relationship” between parts (i.e. asynchronies). An excerpt of a short piano duet by Ottorino Respighi was selected to serve as stimulus material. The duet presents a clear (objective) hierarchical structure, as the melody remains within one part and the accompaniment within the second part of the duet. In this way, the part containing the melody would be said to be structurally leading globally across the whole excerpt, while the second part with the accompaniment would be described as structurally following globally [1], [27]. The (subjective) structural relationship was then manipulated by either cueing participants to attend to the melody part or the accompaniment part of a stimulus (Fig. 1C). The auditory cue involved gradually fading in over five seconds the duet part that was not to be attended. Each stimulus was 25 seconds long in total. Participants were thus cued to listen to the part which was presented (first) from the beginning.

The two kinds of stimuli used varied in the temporal relationship between duet parts (Fig. 1B). One stimulus was a recording of a live performance of two pianists playing the excerpt of the duet. This performance stimulus (which was also described in our previous article [9]) included natural tempo variations within but even more so between the two musicians as well as natural variations in loudness and articulation (degree of overlap and separation between successive sounds). Despite the local temporal leader-follower fluctuations, the recording chosen had no global temporal leader across the performance (i.e., the median asynchrony was 16 ms). The synthetic stimulus entailed a constant velocity of midi velocity 72, which was the average velocity of the melody, while the average midi velocity of the accompaniment in the performance stimulus was 69. Melody and accompaniment velocity rates did not differ significantly (t(1909) = 1009, p = 3.13, n.s.).

The performance was recorded using maxMSP and saved in Musical Instrument Digital Interface (MIDI) format before correcting for small performance errors and omissions in Finale®. Stimuli were then saved as .wav files using the Finale® Grand Piano Timbre.

The second stimulus (which was unique to the current study) was a synthetic, computer-generated metronomic version of the same excerpt. It was produced by entering the sheet music into Finale® and creating a synthetic auditory stimulus without any inter- or intra-part temporal variability. This stimulus therefore contained zero asynchronies, but otherwise the stimulus material was the same as in the performance stimulus (i.e. in terms of pitches, rhythmic categories, and timbre).

The factorial design thus was made up of four conditions: (a) Attend melody in Performance, (b) Attend Accompaniment in Performance, (c) Attend Melody in Synthetic stimulus, and (d) Attend Accompaniment in Synthetic stimulus.

### 2.3 Procedure

The task in each condition of this study required participants to listen and attend to the duet stimulus and then to make several judgments about it afterwards. Written instructions were given to participants before the scanning and task procedure explaining the attention task and the ratings in detail. It is important to note that the two leader-follower factors of structural and temporal relationship were not mentioned in these instructions but that participants were only asked for an assessment of the relationship between parts. Ultimately, participants had to assess (1) the leader-follower relationship of the part to which they had just attended (relative to the other part), (2) the overall performance quality (this was not intended as an emotional judgment but rather as a rational aesthetic and expertise judgment) and (3) the difficulty of the task for the current (just heard) stimulus. Only two of these three possible judgments were required after each stimulus presentation. The order of conditions and ratings was randomized. Each rating was giving within an 8 second time window on a visual analogue scale, which was subsequently converted to an 11-point Likert scale. During the experiment, the different rating scales were labeled with a title “*relationship*” (“Verhältnis”) with the two anchors “leading” (“anführend”) and “following” (“folgend”)(without instructing on any structural or temporal meaning), “*difficulty*” (“Schwierigkeit”) with the anchors “easy” (“leicht”) and “very hard” (“sehr schwer”) and “*quality*” (“Qualität”) with the two anchors “good” (“gut”) and “poor” (“schlecht”). The highest value (10) represented “leading”, “very hard” and a “good” performance, respectively. Responses were given using a button box with two keys. The curser was moved by either single or continuous presses by the index and middle finger of the right hand. A short pre-scan practice trial allowed each participant to get acquainted with the rating scales as well as the time constraints and the response device before the experiment started.

Each stimulus was repeated nine times across the experiment in a randomized order. The trials began with a white fixation cross in the center of the screen for 10–12 seconds, during which time participants were instructed not to react. With the presentation of each stimulus, the fixation cross changed to green. After stimulus presentation the fixation cross changed to white again for 11 seconds before the first of the two Likert-scales appeared, cued by its heading (“relationship”, “difficulty”, or “quality”). Each participant had previously practiced listening to and rating the stimuli in a pilot study, and they were thus familiar with the task. The written instructions provided before scanning ensured that participants understood what they were required to do, as confirmed in a post-scan questionnaire. The experiment was controlled using Presentation software from Neurobehavioral Systems (http://www.neurobs.com/). Stimuli were presented using a specialized audio system (MR-Confon http://www.mr-confon.de/en/) at 80 dB.

### 2.4 Imaging

Magnetic resonance imaging was conducted using a Siemens 3-T Tim trio scanner and a standard bird cage head coil. An echo-planar pulse sequence with a repetition time (TR) of 2000 ms, time to echo (TE) of 28 ms and a 3×3×3 mm^3^ in-plane resolution was used continuously throughout the whole experiment. High resolution t1-weighted images with a resolution of 1×1×1 mm^3^ were used for individual overlays.

### 2.5 Data Analysis

The data analyzed here include those from two of the conditions (Attend melody in Performance & Attend Accompaniment in Performance) reported in our previous article [9] in addition to data from two new conditions (Attend Melody in Synthetic stimulus & Attend Accompaniment in Synthetic stimulus).

#### 2.5.1 Behavioral data

Behavioral ratings were averaged across participants per condition and subjected to a repeated measures analysis of variance (ANOVA). Furthermore, post-hoc t-tests were conducted at an α-level of 0.025 (corrected for multiple comparisons) in order to unpack and establish the directions of effects identified by the ANOVA. All analyses were calculated using SPSS.

#### 2.5.2 Imaging data

Analysis of all neuroimaging data sets was performed using FEAT (FMRIB Expert Analysis Tool) Version 5.98, part of FSL (FMRIB's Software Library, www.fmrib.ox.ac.uk/fsl). Pre-statistic processing included: motion correction using MCFLIRT (Motion Correction FMRIB's Linear Image Registration tool, [35], non-brain removal using BET [36], spatial smoothing using a Gaussian Kernel of 5 mm full width at half-maximum and non-linear high pass temporal filtering (Gaussian-weighted least-squares straight line fitting, with sigma = 40.0 s). Registration included co-registration of the functional scan onto the individual T1 high-resolution structural image and then registration onto a standard brain (Montreal Neurological Institute MNI 152 brain) using FLIRT (FMRIB's Linear Image Registration Tool [35]. Statistical analysis at the individual subject level was carried out using a general linear modeling (GLM) approach [37]. Time-series statistical analysis was carried out using FILM (FMRIB's Improved Linear Model) with local autocorrelation correction [38]. This analysis method allows for incorporation of variance within session and across time (fixed effects) and cross session variances (random effects). Cluster thresholding was performed with a Z-threshold of 2.3 and a corrected p-value of <0.05 with a cluster-based correction for multiple comparisons using Gaussian Random Field Theory [39], [40]. Paired t-tests contrasted the different conditions to explore the effects of structural and temporal relationship.

## Results

In the following, we will describe the results for the 2×2 design with the factors i) Structural Relationship and ii) Temporal Relationship (Fig. 1A). The factors were manipulated by directing attention to either melody or accompaniment and by comparing responses to either a recording of a performance with natural inter and intra-part temporal variations or a synthetic, metronomic stimulus without any temporal variance.

### 3.1 Behavioral results

**Figure 2:**
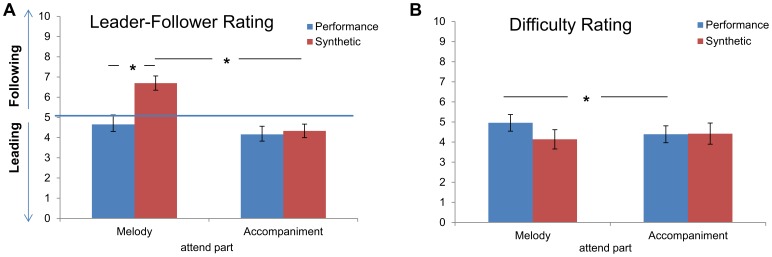
Behavioral ratings. (A) Group mean subjective leader-follower relationship ratings (and standard errors) for Attending to Melody on the left and Attending to Accompaniment on the right. Blue bars represent listening to the performance stimulus, red bars represent the synthetic stimulus. Values >5 (above the horizontal blue line) indicate that the attended part is judged to be subjectively leading, and ratings <5 (below the horizontal blue line) indicate it to be following. (B) Group mean difficulty ratings for all four conditions (and standard error). Asterisks indicate significant differences (α<.025).

Results for the judgment of the leader-follower relationship between parts showed a bias of the structural relationship for melody in the temporally fused synthetic relative to the temporally separated performance stimulus (Fig. 2A). The 2×2 ANOVA for the factors of Structural Relationship and Temporal Relationship on the leader-follower rating data yielded a significant main effect for the Structural Relationship (*F*(1,16) = 9.338, p<0.05) and a significant main effect for the factor Temporal Relationship (*F*(1,16) = 7.223, p<0.05). Furthermore the interaction of both factors was significant ((*F*(1,16) = 9.336, p = <0.05). Post-hoc paired t-tests confirmed that only the Attend to Melody in Synthetic stimulus condition was significantly judged as leading (*t*(16) = 4.85, p<0.001). Comparing the average rating of this condition with Attend to Melody in Performance stimulus (*t*(16) = 3.014, p<0.05) and comparing it with Attend to Accompaniment in Synthetic stimulus (*t*(16) = 3.913, p = <0.05) yielded significant results, confirming the effect.

The difficulty ratings for the four conditions revealed an inverse melody bias for the performance stimulus in relation to the synthetic stimulus (Fig. 2B). A 2×2 ANOVA (Structural Relationship vs. Temporal Relationship) revealed no significant main effect but a significant interaction *F*(1,16) = 4.67, p<0.05. Although none of the post-hoc t-tests were significant when correcting for multiple comparisons (α = .025), these results suggest that the performance stimulus, with its complex temporal relationship, was judged as more difficult, but only when the melody was prioritized.

### 3.2 Imaging results

**Figure 3:**
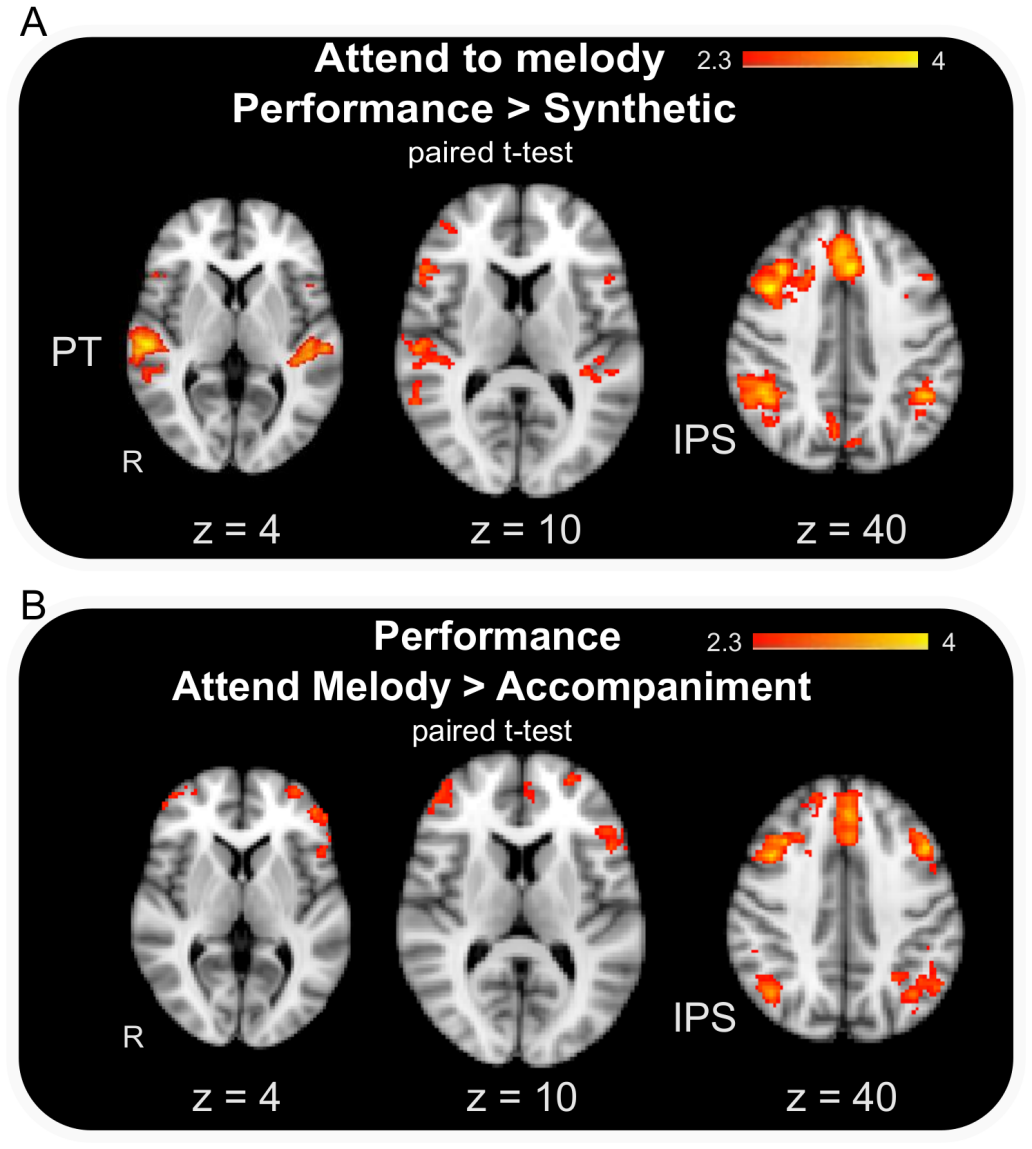
Group mean contrast (mixed effects; Z = 2.3, P = 0.05 corrected) for (A) attending to melody in the performance stimulus relative to the synthetic stimulus and (B) when listening to the performance stimulus attending to melody relative to attending to accompaniment.

Contrasting attend melody conditions with attend accompaniment conditions, as well as performance stimulus conditions with synthetic stimulus conditions, showed a clear BOLD activation bias for the condition in which participants attended to melody in the performance stimulus (Fig. 3 A–B). Only contrasts including this condition revealed significant BOLD activation maps. To explore the effect of varying the temporal relationship within performed multi-part music, we contrasted the conditions Attend melody in Performance stimulus with Attend to Melody in Synthetic stimulus. We found a pattern of BOLD activation which included left superior temporal gyrus, bilateral dorsolateral prefrontal cortex and left inferior frontal cortex in addition to temporal areas, such as the PT bilaterally. The activation map also included right and left inferior parietal areas and midline parietal areas (for details see Table 1A). The reverse contrast showed no significant differences.

**Table 1:** Brain regions that showed significant BOLD activity in the paired t-test contrasts for the (2×2) structural×temporal relationship design.

Anatomical Structure	x,y,z	Coordinates		Z-score
**(A) AttendMelody: Performance >Synthetic**				
L Superior Frontal Gyrus	−2	24	42	3.94
	−6	14	46	3.09
R Middle Frontal Gyrus	42	12	40	4.03
R Dorsolateral Prefrontal Cortex	36	24	40	3.77
	42	32	22	3.58
	46	26	28	3.45
L Dorsolateral prefrontal Cortex	−46	22	26	3.52
L Inferior Frontal Gyrus	−38	18	22	3.01
(Pars Opercularis)				
R Planum Temporale	58	−22	4	3.99
L Heschl's Gyrus	−48	−22	2	3.69
L Planum Temporale	−58	−24	4	3.41
R Intra Parietal Sulcus	46	−46	42	3.77
R Inferior Parietal Lobule	52	−48	50	3.69
L Intra Parietal Sulcus	−44	−48	40	3.65
L Inferior Parietal Lobule	−52	−48	48	3.06
	−48	−54	50	2.88
	−42	−58	42	2.71
R Precuneus	6	−72	46	3.13
	8	−62	38	3.11
L Precuneus	−2	−76	38	2.73
				
**(B) Performance: Attend Melody >Accompaniment**				
R Superior Frontal Gyrus	2	32	32	3.56
L Superior Frontal Gyrus	−2	38	42	3.59
L Dorsolateral Prefrontal Cortex	−44	18	38	3.69
	−38	2	56	3.35
	−34	56	−2	3.62
	−26	56	12	3.05
L Ventrolateral Prefrontal cortex	−48	48	−6	3.19
	−48	46	2	3.12
L Inferior Frontal Gyrus	−50	20	8	3.03
(Pars Opercularis)				
R Inferior Parietal Lobule	42	−62	38	3.52
	22	−70	52	3.02
R Intra Parietal Sulcus	40	−58	54	2.88
L Intra Parietal Sulcus	−44	−54	34	3.35
L Inferior Parietal Lobule	−54	−58	36	3.33
	−48	−62	46	3.25
	−38	−64	42	3.08
R Cerebellum Crus II	36	−78	−38	3.25
L Cerebellum Crus I	−10	−84	−26	3.19
	−36	−80	−28	3.07

(A) Effect of temporal relationship (i.e. natural temporal variations). Significant anatomical regions for attend melody in performance stimulus>attend melody in synthetic stimulus. (B) Contrast of attend to melody>attend to accompaniment in the performance stimulus. Effect of structure. Cluster thresholding was performed with a Z-threshold of 2.3 and a corrected p-value of <0.05 with a cluster-based correction for multiple comparisons using Gaussian Random Field Theory.

In order to examine the effect of musical structure, the conditions Attending to Melody and Attending to Accompaniment during the performance stimulus were contrasted. Attending to Melody in the Performance stimulus yielded significant activation patterns in a fronto-parietal network. Widespread frontal areas were recruited, including right and left superior frontal gyrus, left dorsolateral prefrontal cortex, left ventro lateral prefrontal cortex and left inferior frontal gyrus together with inferior parietal areas and the cerebellum (see Table 1B).

As predicted, we found significant activation within the PT for the performance (i.e. the temporally separated) stimulus relative to the temporally fused synthetic stimulus when attending to melody, whereas the reverse contrast revealed no significant activations. Furthermore, inferior parietal regions were involved in both kinds of contrasts, indicating an involvement beyond the factorial manipulation for the assessment of the relationship between parts (i.e. independent of temporal and structural relationship).

## Discussion

The present study used a paradigm for prioritized integrative attention [23]–[26] in order to investigate the role played by the planum temporale and the inferior parietal cortex in stream segregation and integration during perception of complex auditory stimuli such as music [11]. Participants were cued to attend to one part (melody or accompaniment) of a piano duet and thus had to segregate the streams and keep them separate. A post-listening judgment of the relationship between parts, however, necessitated listeners to concurrently integrate the two parts. In order to explore the underlying mechanisms of segregation and integration, the duet stimuli either had natural temporal variations between parts, or were created synthetically by a computer and were thus metronomic and devoid of asynchronies between tones within and between parts.

Behavioral results suggest a differential influence of the structural (melody vs. accompaniment) and temporal (local asynchronies vs. no asynchronies) factors. The corresponding imaging results identify a large scale network of frontal and parietal areas, including midline structures, often reported during attention to music [11], [34], [41] and attentional tasks in general (eg. [42]).

### 4.1 Behavioral Results

As expected, the subjective assessment of the leader-follower relationship between parts clearly shows an interaction of the structural hierarchical relationship with the temporal relationship factor [11]. Despite the lack of a temporal difference between parts, when attending to the melody in the synthetic stimulus participants rated the attended part as leading. The lack of temporal information meant that the assessment of the leader-follower relationship had to be determined based on the structural hierarchy alone. One could have expected attention to bias the leader-follower relationship rating in favor of the prioritized stream (melody vs. accompaniment) being perceived as more leading, but interestingly this was not the case. Instead, participants judged the synthetic stimulus based only on the structural relationship.

For the temporally separated performance stimulus, both the structural and the temporal relationship had to be considered. It is interesting to note that based on the behavioral data the performance stimulus was rated solely on the temporal relationship between parts. The performance stimulus had no global temporal leader, but as seen in the plot of asynchrony between parts over time (Fig. 1B), this stimulus had natural local fluctuations, which meant that the duet parts temporally exchanged leader-follower roles repeatedly across the length of the stimulus. It seems plausible that the attentional and working memory demands when monitoring these temporal fluctuations led to an overestimation of the temporal relationship or a suppression of the importance of the structural relationship as a factor for the leader-follower relationship. Timing tasks are very sensitive to disruptions by secondary tasks and even small increases in cognitive load [43]. It is therefore effortful to keep track of temporal modulations such as those associated with the asynchronies in our performance stimulus. Nevertheless, the data did not yield evidence for overestimation or suppression of the second relationship factor. Future studies could include a question about the weight assigned to the two factors when making the ratings to guide the process of making such interpretations. In this study, the main focus was on the influence of the two factors without the specific instruction of basing ratings on these two factors.

The influence of both factors can be investigated with the subjective difficulty estimations of the conditions. Here, the performance stimulus was rated as more difficult, specifically when attending to melody, pointing to an interaction of both relationship factors on the level of cognitive load. (It should however be noted that the range of ratings across all four conditions was particularly small: 4–5; see Fig. 2B) We had expected that, due to the highly variable temporal information in the performance stimulus, this natural stimulus might be perceived as harder to prioritize, requiring intensive segregation and monitoring of the two streams, thus increasing cognitive load. If the temporal fluctuations however were the factor driving the difficulty rating, there should be no difference when manipulating the attended part. However, an effect was only seen in the Attend to Melody condition, suggesting a structural bias and thus an interaction of both leader-follower relationship factors. One might speculate that the lack of influence we found for the factor of structural relationship in the ratings of the leader-follower relationship for the performance stimulus might have led to the subjective feeling of the performance stimulus being harder to judge when attending to melody. The bias in the difficulty rating when attending to melody could thus also be partially do to the awareness of an overestimation of the temporal relationship factor in the relationship judgment for the performance stimulus. The interaction of both relationship factors nevertheless, leads us to conclude that participants actually attended to and integrated both streams.

### 4.2 Planum Temporale and Segregation

As predicted, we found significant activation within the PT when comparing the performance (i.e. the temporally complex) and the synthetic (i.e. temporally simple) stimuli when the melody was attended. No such activation was observed in a contrast of the manipulated attention conditions (melody vs. accompaniment) for the performance stimulus. This suggests that the activation of the PT was not due to attending to melody per se but rather to the different temporal segregation qualities of the performance stimulus in contrast to the synthetic stimulus. This activation is in line with recent findings that the PT is involved in the stream segregation process [12], [13], [44], [45]. The evidence suggests that object properties such as the spatial position of the auditory source, as well as other grouping cues, are used within the PT in order to segregate incoming auditory streams and forward this information about the streams to association cortices for further analysis [12], [13], [44]. In a recent fMRI study [13], participants were required to listen to stimuli which either consisted of one talker or three talkers. Those talkers appeared at the same location, at different locations, or appeared to be moving in space. Results showed that PT activation was directly modulated by the spatial manipulation of the stimuli, with a higher BOLD signal for the more complex three spatial positions compared to the simpler single spatial position. Nevertheless, the results also indicated that PT activity was directly related to the number of streams present in the stimulus. The authors were thus able to show that PT activation is modulated not only by spatial properties or varying spatial properties of streams but also by the number of streams to be separated. The present study points to a similar modulation influenced by the temporal properties of the streams. Our performance stimulus, which included natural performance asynchronies and thus a temporal deviation between sounds, could be segregated based on these complex temporal cues. The synthetic stimulus on the other hand was not differentiable by temporal cues and thus resulted in less effortful perception and judgments. This finding is consistent with studies indicating superior temporal sulcus involvement in temporal discrimination [14], [15]. Kanai and colleagues [14] down-regulated the auditory and the visual cortex in turn in order to test whether early sensory cortices contribute to time processing in general or whether their contribution was modality specific. Results showed that the disruption of the visual cortex only impaired visual temporal processing whereas the disruption of the auditory cortex impaired both visual and auditory temporal processing. The authors argue that this dissociation is due to greater temporal resolution of the auditory sensory cortex, which makes it a prime candidate to process time independently of modality [14].

The contrast of attending to melody in the performance stimulus relative to the synthetic stimulus (Fig. 3A) clearly shows greater activation of the PT for the performance stimulus, as might be predicted by the modulation of PT activation by complexity (i.e. the number of streams or spatial positions in [13]). The present results thus broaden the spectrum of stream attributes that may be used by the PT in order to segregate streams, and point to a more general function of stream segregation based on stimulus or stream properties. In line with this proposal, the contrast of attending to melody in the performance stimulus relative to attending to accompaniment in this stimulus (Fig. 3B) shows no significant PT BOLD activation. It could be suggested that a negative result is due to the fact that, in the performance stimulus, the same degree of temporal complexity is present whether one is attending to the melody or the accompaniment. The same applies for the temporal cues, which are the same irrespective of the part that one prioritizes. In this way, it seems that the PT activation is modulated in the same manner for the performance stimulus independently of the structural relationship.

### 4.3 Inferior Parietal Cortex and Integration

The IPC also is another key structure that has been suggested to play a role in processes involved in auditory temporal perception [19]–[22], [46]. More specifically, down-regulating the right IPC with repetitive TMS pulses impairs auditory temporal order judgments [21]. Such judgments were necessary in the present study for the assessment of the temporal relationship between parts and were thus involved in the temporal leader-follower relationship. Existing evidence, however, not only points to IPC involvement in auditory time perception but in time perception in general [20]. Comparing visual and auditory time perception, Bueti and colleagues measured MT/V5 and IPC activation in the context of time duration discrimination tasks with auditory and visual stimuli as well as a visual spatial task. These authors found that MT/V5 plays a role in temporal as well as spatial processing only in the visual modality, while the IPC seems to be involved in multimodal processing of time ([20]; see also [47]). It should be noted that studies of time perception have primarily indicated involvement of the right IPC whereas the present results show bilateral activation of the IPC in both contrasts.

The second part of our task, the assessment of the leader-follower relationship between parts, necessitated participants not only to segregate the concurrent streams but additionally to integrate them in a common representational space in order to assess the relationship between them. As participants were not instructed specifically about the two leader-follower factors manipulated in this study, the assessment of the relationship most likely incorporated both factors on a neural level, namely the temporal and structural relationship [11]. Zatorre and colleagues recently proposed that the IPS is involved in computing the relationship between stimulus elements [10]. In their task, they had subjects reverse melodies, paralleling work on mental rotation in the visual domain. These authors also found greater IPS activation for the reversal of melody than when participants had to listen to and retain the regular melodies (forward melody condition). In mental rotation studies, a similar BOLD response dependency has been shown, as the percent signal change in the IPS depends on the degree of mental rotation necessary for the task [48], [49]. The role of the IPS seems thus to be a more general one related to computing the degree of the relationships between items independent of the kind of relationship.

Furthermore, the assessment of the leader-follower relationship connects the IPS with a general integration process. The task of judging leader-follower relations on a scale requires the relationship between parts to be assigned a magnitude or a distance value. More specifically, the dorsolateral prefrontal cortex and the intraparietal sulcus (IPS) have been implicated in the monitoring and the manipulation of information held in working memory [50]. In the present study, this activation could therefore be due to holding the two streams separate within the dlPFC [51] and assessing the relationship between them within the IPC [9], [16].

In line with its hypothesized role in calculating the relationship between stimulus elements, it has been proposed that the IPC is central to magnitude estimations for different kinds of stimuli within different reference frames (eg. spatial and temporal) [9], [16], [17]. Walsh [16] concludes that the IPC is implicated in tasks that involve space, time and quantity. Such information about attributes that are informative about the relationship between objects is needed in order to interact with the external world through coordinated actions.

The IPC activation in the present study might nevertheless not be purely based on the magnitude estimation of temporal attributes of the stimuli. Both contrasts (see Fig. 3 A–B) point to an influence of the structural relationship factor on IPS activation. The structural relationship, or a dominance of melody, was predicted to influence the leader-follower assessment [11]. The fMRI results indicate that the IPS is influenced by structural relationship, as IPS activation becomes significant for attending to melody in the performance stimulus compared to the synthetic stimulus and compared to attending to accompaniment. This is noteworthy as it points to a difference between the influence of the structural relationship factor on the two processes of segregation and integration. Recent studies from the visual domain report top-down modulatory effects and an associated increase in BOLD response [52], [53]. In fact, those studies report that the modulatory effect involves a similar network including the IPC and frontal regions [52], [54], [55]. A learned structural hierarchy of melody and accompaniment within western music may explain the observed IPS activity that is indicative of integration processes implemented when listening to the performance stimulus.

### 4.4 Implications and Conclusion

Musicians' abilities to segregate concurrent musical streams which are similar in timbre and harmony and thus pulled towards vertical fusion instead of horizontal separation also have clinical applications. Studies examining musicians' abilities have shown that musicians are better at segregating streams [56] and better at detecting speech in noise compared to non-musicians [57], [58]. This enhancement of sensory and cognitive abilities might be due to superior segregation mechanisms for auditory streams and top-down feedback mechanisms, which enable relevant acoustic features to be enhanced in early sensory processing stages ([59], [60] for a review see [61]). Moreover, such mechanisms and abilities are of interest to research on ageing [62], temporal processing disorders [60], but also more generally for research on complex auditory scene analysis. In summary, multi-part music, as a model, not only sheds light on the neural underpinnings of segregation and integration processes involved in listening to music, it also extends knowledge about general auditory scene analysis. The requirement of prioritized integrative attention combined with a relationship judgment provides a novel paradigm that necessitates both segregation and integration, and allows the influence of different kinds of elementary relationships to be explored.
